# Relevant Measures to Prevent the Spread of African Swine Fever in the European Union Domestic Pig Sector

**DOI:** 10.3389/fvets.2018.00077

**Published:** 2018-04-16

**Authors:** Cristina Jurado, Marta Martínez-Avilés, Ana De La Torre, Marina Štukelj, Helena Cardoso de Carvalho Ferreira, Monica Cerioli, José Manuel Sánchez-Vizcaíno, Silvia Bellini

**Affiliations:** ^1^VISAVET Health Surveillance Centre, Animal Health Department, Veterinary Faculty, Complutense University of Madrid, Madrid, Spain; ^2^Animal Health Research Centre, National Institute for Agricultural and Food Research and Technology (INIA-CISA), Madrid, Spain; ^3^Veterinary Faculty, University of Ljubljana, Ljubljana, Slovenia; ^4^GD Animal Health Research Institute, Deventer, Netherlands; ^5^Istituto Zooprofilattico Sperimentale della Lombardia ed Emilia Romagna (IZSLER), Brescia, Italy

**Keywords:** biosecurity, Europe, epidemiology, pig farm, preventive measures

## Abstract

During the past decade, African swine fever (ASF) has spread from the Caucasus region to eastern European Union countries affecting domestic pig and wild boar populations. In order to avert ASF spread, mitigation measures targeting both populations have been established. However, despite these efforts, ASF has been reported in thirteen different countries (Georgia, Azerbaijan, Armenia, the Russian Federation, Ukraine, Belarus, Estonia, Latvia, Lithuania, Poland, Moldova, Czech Republic, and Romania). In the absence of an effective vaccine or treatment to ASF, introduction and spread of ASF onto domestic pig farms can only be prevented by strict compliance to control measures. This study systematically reviewed available measures to prevent the spread of ASF in the EU domestic pig sector distinguishing between commercial, non-commercial, and outdoor farms. The search was performed in PubMed and using a common browser. A total of 52 documents were selected for the final review process, which included scientific articles, reports, EU documents and official recommendations, among others. From this literature review, 37 measures were identified as preventive measures for the introduction and spread of ASF. Subsequently, these measures were assessed by ASF experts for their relevance in the mitigation of ASF spread on the three mentioned types of farms. All experts agreed that some of the important preventive measures for all three types of farms were: the identification of animals and farm records; strict enforcement of the ban on swill feeding; and containment of pigs, so as to not allow direct or indirect pig–pig and/or pig–wild boar contacts. Other important preventive measures for all farms were education of farmers, workers, and operators; no contact between farmers and farm staff and external pigs; appropriate removal of carcasses, slaughter residues, and food waste; proper disposal of manure and dead animals, and abstaining from hunting activities during the previous 48 h (allowing a 48 h interval between hunting and being in contact with domestic pigs). Finally, all experts identified that the important preventive measures for non-commercial and outdoor farms is to improve access of those farms to veterinarians and health services.

## Introduction

African swine fever (ASF) is an infectious disease of swine notifiable in the European Union (EU) and to the World Organization for Animal Health (OIE). Susceptible pigs can be infected by direct or indirect contact with infectious animals or their fluids, ingestion of contaminated animal feed, pork, or pig products, or contact with contaminated surfaces or fomites (clothing, footwear, vehicles, farming tools, etc.) acting as mechanical vectors (1). In the southern and eastern parts of the African continent and the Iberian Peninsula, ASF can also be transmitted by biological vectors, infected soft ticks belonging to the *Ornithodoros* genus (2). No vaccine or treatment is available against ASF. Therefore, prevention and control of the disease is mainly based on the early detection of the disease by timely recognition in the field and efficient laboratory diagnosis, followed by the implementation of strict sanitary measures (2–4). Adequate implementation of sanitary measures will reduce the number of secondary outbreaks on domestic pig farms, which will decrease the potential contamination of the environment and, finally minimize the likelihood of infection in wild boar (5).

Since 1978 and until recently, the Italian island of Sardinia has been the only European ASF-infected area (6). However, in 2007, ASF was introduced into Georgia, from there it spread to neighboring countries Azerbaijan and Armenia. As a result of the disease introduction and spread throughout the Russian Federation and Belarus, the EU strengthened its preparedness against ASF. Among the protection measures implemented by EU member countries bordering the Russian Federation were actions such as improving cleaning and disinfection of livestock vehicles, suspension of livestock markets, surveillance, enhanced biosecurity on farms, and awareness campaigns. Moreover, contingency plans were revised and the diagnostic capabilities of the EU labs were assured. However, ASF entered into four EU member countries in 2014, namely Lithuania, Poland, Latvia, and Estonia; and in 2017, ASF was reported for the first time in Czech Republic and Romania (7). During this period, between January 2014 and December 2017, ASF outbreaks (occurrence of one or more ASF cases on a pig farm) were reported in over 250 farms, and more than 8,500 wild boar cases (an individual wild boar infected by ASF virus) were reported within the EU (7–10). As a reaction to this large number of outbreaks and cases, the Community Veterinary Emergency Team recommended several measures such as: (i) focus surveillance on wild boar and domestic pigs, (ii) implement control of animal movements, (iii) safe disposal of wild boar carcasses, (iv) avoid swill feeding practices, (v) implement biosecurity on farms, (vi) conduct awareness campaigns and finally, and (vii) review wild boar hunting practices (11). These measures were aimed at reducing the risk of spread of the disease to domestic pig farms and its transmission between wild boar populations. In contrast to what has been observed in non-EU European countries (i.e., the Russian Federation or Ukraine), in the EU scenario the number of infected farms has been comparatively lower, with wild boar being the most severely affected host (7, 8).

The main piece of legislation providing the tools for the control of ASF in the EU is the Council Directive 2002/60/EC (9), which establishes the minimum measures to be applied within the EU for the control of ASF. It includes the measures to be taken in the event of an outbreak of ASF on a pig holding and in cases where the disease is suspected or confirmed in feral pigs. The main objectives of controlling ASF in feral pigs are to reduce the risk of transmission to domestic pigs and to prevent it becoming endemic in the feral pig population (see Definitions) (9). The Directive lays down the measures to be taken in the infected area and the provisions to apply on the holdings of that area. All control and eradication measures applicable are based on classical disease control methods, which include surveillance, epidemiological investigation, tracing of pigs, and stamping out in infected holdings. These measures are applied in combination with strict quarantine and biosecurity measures on domestic pig holdings and animal movement control. The Directive also requires that Member States develop and implement plans for the eradication of the disease.

Moreover, specific regionalization measures are laid down in Commission Implementing Decision 2014/709/EU (10). This Decision establishes animal health control measures on the movement, dispatch of pigs and certain pig products, and marking pig meat from the areas at risk of infection in order to prevent the spread of ASF to other areas of the Union. Affected Member States and territories are listed in different parts of the Annex to the Decision, the differentiation is made based on their epidemiological situation and level of risk. The Annex is divided into four parts, and territories that are listed in Part IV have a higher risk of spread of ASF than the ones listed in Part I. In determining the application of control measures on a certain commodity of a certain territory, the level of risk of that area and the type of commodity is taken into account. Indeed, in terms of risk of spread of ASF, movement of different porcine commodities poses different levels of risk. It is worth to mentioning that this Decision is also aimed at avoiding unnecessary disturbance to trade within the EU, as well as avoiding unjustified barriers to trade by third countries and the provisions that are set in this Decision are aligned with the OIE standards (11).

Bearing in mind all of the above, the aim of this study is to review described measures to prevent the introduction and further spread of ASF in the domestic pig sector focused on the EU scenario. An additional aim of this review was to assess the importance of these identified measures depending on the different pig farming systems (see materials and methods section). Adequate identification of relevant measures will allow for the creation of guidelines for pig producers to prevent the spread of ASF, which is one of the identified goals of the COST Action 15116 Understanding and combating African swine fever in Europe (ASF-STOP) supported by COST (European Cooperation in Science and Technology).

## Materials and Methods

### Literature Sources and Search Strategy

Following an approach similar to Rodríguez-Prieto et al. (12), the systematic review targeted preventive measures to avoid the spread of ASF in the domestic pig sector described in scientific publications, gray literature (materials produced by organizations outside the academic publishing channels), technical guidelines and international, national, and regional regulations. The literature search was performed in 3rd March 2017 and supplemented with further search in 14th December 2017 using PubMed database^1^ for scientific articles. Scientific papers written in English (for reviewing convenience) between the last 39 years (1978 and 2017) were reviewed. A list of key words was combined into a Boolean query to identify titles and/or abstracts of documents of interest. The key words used (and any word containing the stem presented) were “African swine fever,” “Preventive measure/s,” “Biosecurity,” “Risk,” and “Pig farm.” The search terms applied were “African swine fever” AND [Preventive measure* OR Biosecurity OR Risk OR Pig farm]. To make sure other relevant documents such as technical guidelines, regulations, or scientific opinions, among others, were included, the literature search was performed following the same query on the internet using a common browser.

### Definitions

“*Control measures*” are defined as the best/safest options to eliminate or reduce specific risks, while “*preventive measures*” are actions taken to avoid specific risks (13). As the glossary of the Terrestrial Animal Health code of the OIE states (14), “*biosecurity*” means a set of management and physical measures designed to reduce the risk of introduction, establishment, and spread of pathogenic agents to, from and within an animal population. On the other hand, “*risk*” means the likelihood of the occurrence and the likely magnitude of the biological and economic consequences of an adverse event or effect to animal or human health (14).

Based on the working document SANTE/7113/2015-Rev 7 produced by the Directorate-General for Health and Food Safety (15) pig farming systems and subsequently, pig farms can be classified as: (i) “*commercial farms*” which refers to farms that sell pigs, send pigs to a slaughterhouse or move pig products off the holding, (ii) “*outdoor pig farms*” which refers to farms in which pigs are kept temporarily or permanently outdoor, and (iii) “*non-commercial farms*” which refers to farms where pigs are kept only for fattening for own consumption and neither pigs nor any of their products leave the holding. Elsewhere, this last type of farm is referred as “*family farms*” (16) or “*backyard farms*” (17). Commercial farms can be divided into multi-site farms which are holdings specialized on one production step (farrowing, nurseries, or finishing) and on-site farms which are premises that produce all production steps (18). Moreover, “*feral pig*” or “*free-ranging pig*” means a pig which is not kept or bred on a holding according to the Council Directive 2002/60/EC (19). In Sardinia, free-ranging pigs are usually referred as “*brado*” (16, 20).

### Study Selection

A two step-process was followed to select the literature relevant for the aim of this review. A primary exclusion criteria was applied when reading title and abstract of found literature (abstract when available): (i) published before 1978; (ii) not related to the theme of this review; (iii) not related to the European scenario; and (iv) repeated document (already selected among retrieved results). If abstract were not available, the piece of literature would be kept for the next stage. Then, the full text of each selected piece of literature was screened. As a second exclusion criteria, documents (v) which full text was not available; (vi) no preventive measures were described; (vii) described preventive measures were not focused on ASF; or (viii) information on the theme was insufficient, were excluded. The explained process was individually performed by three reviewers following the mentioned exclusion criteria in order to cross-check selected literature and resolve any disagreement.

### Assessment of Described Preventive Measures

A group of experts was invited to participate in an expert opinion session to assess the preventive measures identified in this review.

Participants belonging to the COST (European Cooperation in Science and Technology) action: “Understanding and combating African swine fever in Europe” (ASF-STOP) supported by COST (COST Action 15116)^2^ were encouraged to suggest experts with relevant expertise in ASF prevention, ASF control and eradication, ASF epidemiology and the EU domestic pig sector.

Before starting the assessment, the list of measures were reviewed by authors to ensure measures were accurate and clear, as well as no measures were omitted. In total, 20 experts were invited to participate and contacted by email, 12 of them returned their responses.

Experts were asked to assess the relevance of each described preventive measure by answering yes or no to the closed question: “Is this measure important for commercial, non-commercial, and outdoor-farms?” “Importance” was defined as the perceived need for each measure. Experts were asked to perform this assessment within the EU context. Moreover, experts were encouraged to suggest additional measures if they thought they were missing. Results were recorded in an Excel datasheet (Microsoft Corp., Redmond, WA, USA).

## Results

### Selection Process

**Figure 1:**
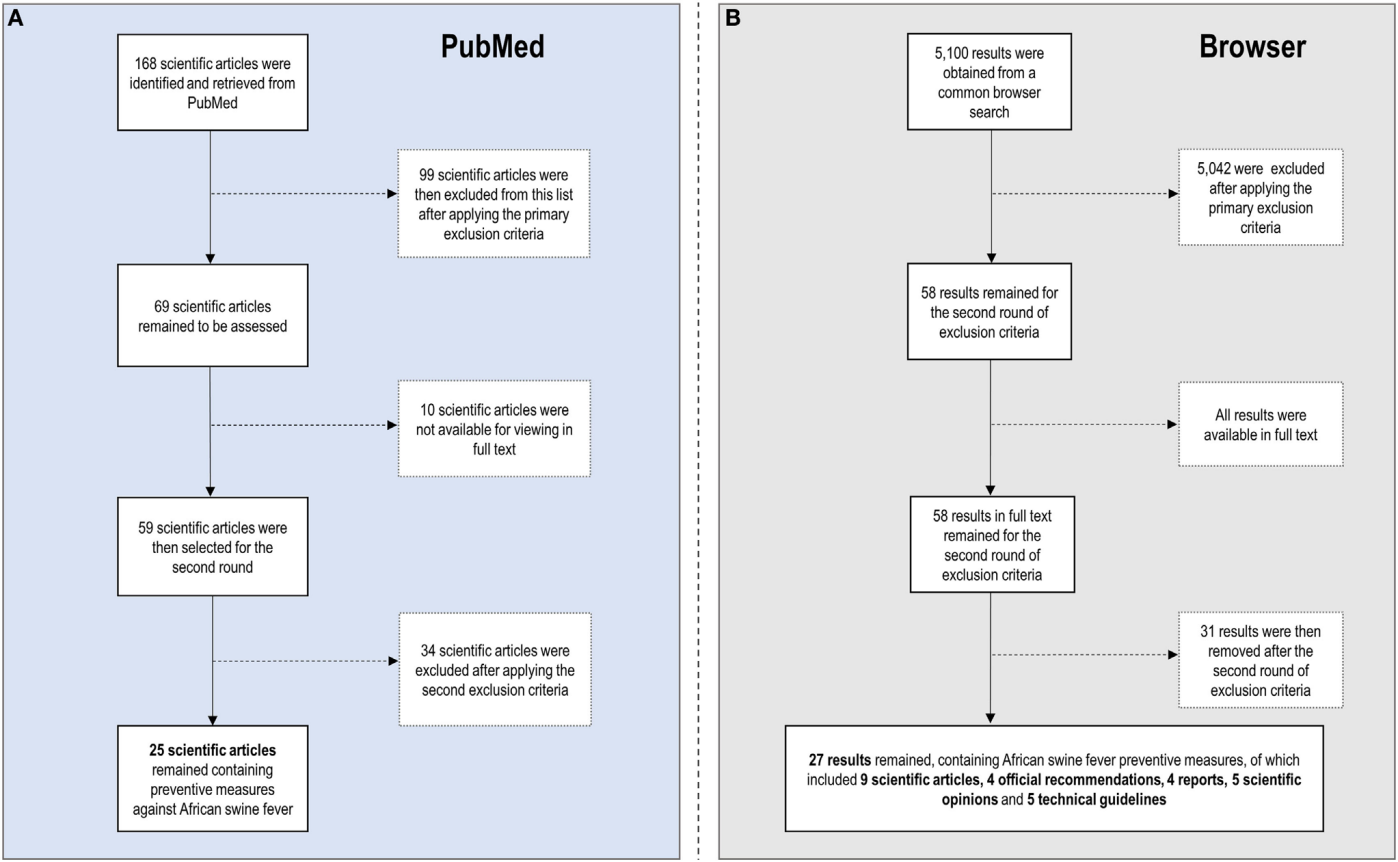
Flowchart summarizing the literature selection process **(A)** on PubMed database and **(B)** on a common browser.

Figure 1 shows the literature selection process and Table 1 compiles the selected literature. The search made on PubMed database returned 168 scientific papers. After applying the primary exclusion criteria, 69 were selected for the second step of the review. However, the full text was not available for 10 of them. Therefore, 59 scientific articles were selected for the second screening round. The same search on a common browser returned 5,100 results of potential interest. By applying the primary exclusion criteria, 58 results were selected for the second round, all of them had available the full text.

**Table 1:** Pieces of literature included in the review process.

ID	Title	Search	Type	Reference
1	African and classical swine fever: similarities, differences and epidemiological consequences	PubMed	Article	(21)
2	Why is African swine fever still present in Sardinia?	PubMed	Article	(16)
3	African swine fever in eastern Europe: the risk to the UK	PubMed	Article	(22)
4	Understanding African swine fever infection dynamics in Sardinia using a spatially explicit transmission model in domestic pig farms	PubMed	Article	(23)
5	Control of African swine fever epidemics in industrialized swine populations	PubMed	Article	(24)
6	Preventive measures aimed at minimizing the risk of African swine fever virus spread in pig farming systems	PubMed	Article	(5)
7	Modelling African swine fever presence and reported abundance in the Russian Federation using national surveillance data from 2007 to 2014	PubMed	Article	(25)
8	English pig farmers’ knowledge and behaviour towards African swine fever suspicion and reporting	PubMed	Article	(26)
9	Simulating the epidemiological and economic effects of an African swine fever epidemic in industrialized swine populations	PubMed	Article	(27)
10	A cartographic tool for managing African swine fever in Eurasia: mapping wild boar distribution based on the quality of available habitats	PubMed	Article	(28)
11	Transmission routes of African swine fever virus to domestic pigs: current knowledge and future research directions	PubMed	Article	(1)
12	Expert opinion on the perceived effectiveness and importance of on-farm biosecurity measures for cattle and swine farms in Switzerland	PubMed	Article	(29)
13	Spatiotemporal analysis of African swine fever in Sardinia (2012–2014): trends in domestic pigs and wild boar	PubMed	Article	(30)
14	Statistical exploration of local transmission routes for African swine fever in pigs in the Russian Federation, 2007–2014	PubMed	Article	(31)
15	Evaluation of the risk factors contributing to the African swine fever occurrence in Sardinia, Italy	PubMed	Article	(32)
16	Spatio-temporal modeling of the African swine fever epidemic in the Russian Federation, 2007–2012	PubMed	Article	(33)
17	Thirty-five-year presence of African swine fever in Sardinia: history, evolution and risk factors for disease maintenance	PubMed	Article	(6)
18	The medical and veterinary role of *Ornithodoros erraticus* complex ticks (Acari: Ixodida) on the Iberian Peninsula	PubMed	Article	(34)
19	Pig producers urged to review biosecurity as ASF and PED spread	PubMed	Article	(35)
20	African swine fever in the North Caucasus region and the Russian Federation in years 2007–2012	PubMed	Article	(36)
21	African swine fever (ASF): five years around Europe	PubMed	Article	(37)
22	African swine fever: an epidemiological update	PubMed	Article	(38)
23	Qualitative risk assessment in a data-scarce environment: a model to assess the impact of control measures on spread of African swine fever	PubMed	Article	(39)
24	Viruses in boar semen: detection and clinical as well as epidemiological consequences regarding disease transmission by artificial insemination	PubMed	Article	(40)
25	Temporal and spatial patterns of African swine fever in Sardinia	PubMed	Article	(41)
26	Do not bring African swine fever to Finland	Browser	Official recommendation	(42)
27	African swine fever facing Romania	Browser	Report	(43)
28	African swine fever	Browser	Official recommendation	(44)
29	African swine fever—Guidance	Browser	Official recommendation	(45)
30	Guidelines for the cost effective prevention and control of African swine fever	Browser	Report	(46)
31	African swine fever in Poland and Baltic countries	Browser	Report	(47)
32	Gaps in African swine fever: analysis and priorities	Browser	Article	(48)
33	African swine fever (ASF)	Browser	Article	(49)
34	African swine fever: new challenges and measures to prevent its spread	Browser	Article	(50)
35	African swine fever	Browser	Scientific opinion	(51)
36	Review of African swine fever: transmission, spread and control	Browser	Article	(52)
37	African swine fever: how can global spread be prevented?	Browser	Article	(4)
38	African swine fever	Browser	Scientific opinion	(53)
39	Epidemiological analyses of African swine fever in the Baltic States and Poland	Browser	Scientific opinion	(10)
40	Role of tick vectors in the epidemiology of Crimean-Congo hemorrhagic fever and African swine fever in Eurasia	Browser	Scientific opinion	(54)
41	African swine fever	Browser	Scientific opinion	(55)
42	Implementation of a regional training program on African swine fever as part of the cooperative biological engagement program across the Caucasus region	Browser	Article	(56)
43	African swine fever in the Caucasus	Browser	Report	(57)
44	African swine fever: detection and diagnosis. A manual for veterinarians	Browser	Technical guideline	(58)
45	African swine fever in wild boar in Europe: a notable challenge	Browser	Article	(59)
46	The costs of preventive activities for exotic contagious diseases-A Danish case study of foot and mouth disease and swine fever	Browser	Article	(60)
47	African swine fever strategy for Eastern part of the EU	Browser	Official recommendation	(15)
48	African swine fever in wild boar and African wild suids	Browser	Technical guideline	(61)
49	Transboundary and emerging viral infections of pigs in central and eastern Europe	Browser	Technical guideline	(62)
50	Guidelines on surveillance and control of African swine fever in feral pigs and preventive measures for pig holdings	Browser	Technical guideline	(63)
51	Good practices for biosecurity in the pig sector	Browser	Technical guideline	(18)
52	New insights into the role of ticks in African swine fever epidemiology	Browser	Article	(64)

After applying the second exclusion criteria and completion of the screening rounds, 34 articles (25 retrieved from PubMed and 9 retrieved from the browser search), 4 official recommendations (meaning information coming from governmental authorities), 4 reports, 5 scientific opinions, and 5 technical guidelines were included in the review. The rest of the documents including reports, recommendations, and guidelines were retrieved from the browser search.

### Results From the Systematic Review

Preventive measures described hereinafter were obtained from the 52 pieces of literature selected during the systematic review. These measures were classified in four different groups: general prevented measures suggested for all types of farms (as some of them were common for commercial, non-commercial, and outdoor farms), and three groups of suggested measures for each of the identified types of farms.

#### General Preventive Measures

The risk of introduction and exposure to ASF depends on the epidemiological characteristics of the country, area, and type of farm (31, 70, 74–80). Pig production in Europe is highly heterogeneous with different biosecurity standards and productive levels (39, 81). Actions to prevent ASF introduction and spread should take into consideration the epidemiology of the disease, with especial focus on the virus resistance in the environment, routes of transmission, and excretion as well as the characteristics of the farming systems in place (5, 29, 38, 48, 58). As no vaccine for ASF is available, prevention of ASF relies upon implementing strict biosecurity measures to avoid potential contact between domestic pigs and ASF virus (35, 49, 58, 82). In the EU, movements of pigs or pig products coming from infected areas have been prohibited to prevent ASF spread (4, 19, 21). Moreover, the presence of infected wild boar in the area and its hunt constitutes an additional source of risk that cannot be discarded (82, 83). Minimum biosecurity requirements to apply during hunting in the affected territories have been proposed (5, 15, 61). First of all, hunters shall be authorized to hunt after receiving training on basic biosecurity practices. Hunted wild boar should be tested and only released after receiving negative results. Hunted animals should be moved to the dressing facilities in dedicated vehicles, private cars should be parked outside the hunting field. Dressing facilities would be used if they have tap water, electricity, freezers, and waste water collection. Evisceration should be performed with gloves at the dressing facilities and hands gently washed with soap and water. Offal should be stored in proper containers in the dressing area and then, cleaned and disinfected. Clothing, footwear, and hunting equipment should be cleaned and disinfect after each use (clothing washed at 60°C). Finally, contact with domestic pigs should be avoided, allowing a 48 h interval between hunting and being in contact with domestic pigs. All of above the needs to be implemented together with education and training campaigns to get hunters involved in control strategies as much as possible (5). Thoen et al. (84) and Sánchez-Vizcaíno et al. (50) also suggested that systems that wild boar can use as artificial feeding places (feeders, water holes, supplementary feeding of ungulates) should be avoided, as these systems can significantly increase wild boar abundance and spatial concentrations. However, it has been also suggested that this ban may be deemed effective only in regions where the habitat is unsuitable for wild boar and where feeding caused artificial population establishment (53).

The EU Commission has established minimum biosecurity requirements for commercial, non-commercial, and outdoor farms (63). Health status and free-ASF certificates have to be checked before acquiring new animals (15, 16, 18, 45, 58, 63, 64). On breeding farms, semen (21, 40, 45), embryos, or ova should come from free-ASFV certified farms (15, 19, 44, 57). Visits should be discouraged (44, 45), limiting access to the farm and animals, to workers and veterinarian services (5, 31, 52, 63). If visitors enter the farm, visits should be registered and visitors should follow strict biosecurity measures regarding footwear and clothing (45, 47, 52, 57, 58, 64). Farm staff should follow the same biosecurity procedures. Likewise, workers and owners should be aware and well trained with regard to ASF (22, 23, 37, 38, 47, 48, 52, 57, 63) as well as veterinarians and operators along the market chain (58). Moreover, farm staff must not have contact with animals from other pig premises nor own pigs (4, 5, 15, 16, 57). In addition to this, the Finnish Food Safety Authority recommends that farm staff should not directly enter the farm after visiting a farm abroad, they should wait at least for 48 h (42).

Regarding physical barriers on farms, animals should be kept in a way that ensures that no direct, nor indirect contact occurs with wild boar, feral pigs, or domestic pigs coming from other premises (15, 63). Additionally, perimeter fences should delimitate the commercial holding to prevent such contact (15). On outdoor farms, fences will be preferably doubled (63), at least 1 m apart (57), and proofed against wild boar and pigs (15, 16, 18, 45, 47, 65). Fences should be at least 2 m high of which 50 cm should be under the ground (66).

In addition, as part of good farming practices, carcasses, discarded parts from slaughtered pigs and food waste should be disposed in accordance with Regulation (EC) No. 1069/2009 (57, 58, 63, 67). Sharing equipment between holdings should be discouraged (45, 47, 52, 55, 57, 63), and footbaths should be used at the entrance of every unit where animals are held (5, 15, 52, 58, 63). Organic material should be removed from footwear prior to disinfecting (49). Animals must be checked at least once a day paying special attention to mortality rates and clinical signs compatible with ASF (45). Moreover, cleaning and disinfection protocols should be established and periodically performed on every farm facility, vehicle, and piece of equipment (15, 16, 18, 35, 42, 44, 55, 57, 63). Disinfectants effective against ASF virus include 2% caustic soda, 2% sodium hypochlorite, 0.3% formalin, 3% ortho-phenylphenol, and iodine compounds, among others (58, 85). Organic material (feces, feed, bedding materials) should be completely removed to maximize the efficacy of disinfection (49).

Moreover, regarding the location of pig farms, several scientific publications point out that farms should be located far from suitable wild boar areas and close to geographical barriers (such as mountains, rivers, etc.) (16, 28, 48, 53). Finally, Mellor et al. (68) observed experimental transmission of ASF through *Stomoxys calcitrans* flies. Therefore, given this potential role of stable flies as mechanical vectors, it has been suggested that sanitation, biological, and chemical controls should be applied to suppress stable flies. As an example, commercial and non-commercial farms could eliminate fly breeding sites in combination with placing insecticide-treated nets to reduce the potential risk posed by flies (45, 69).

Specific preventive measures based on biosecurity have been proposed depending on the type of farm: commercial, outdoor, or non-commercial (15).

#### Specific Measures Focusing on Commercial Farms

Commercial farms are significantly larger in size and number of animals (18) and so, the economic and animal health impact of ASF is greater than on outdoor and non-commercial farms (24, 29).

Key measures to prevent the introduction of ASF on commercial farms are to establish clear clean/dirty areas for personnel including changing rooms and shower (15, 18, 31, 49, 55) and to review logistical arrangement for entry of new animals. This measure will allow for the adequate identification of critical control points (15), which is particularly relevant since contaminated vehicles transporting pigs or carcasses are associated to a high risk of disease transmission (18, 70).

Several steps should be included when preparing a protocol for animal transport. First, farms should be designed to allow deliveries without entering the farm (5, 45, 63). If it is not possible, decontamination of vehicles is necessary before entering the farm (42, 58, 65). Employees involved in pig transport should not come in contact with farm workers nor with animals (5, 45). If other drivers (i.e., animal feed suppliers) need to enter the farm, footwear should be changed, cleaned, and disinfected when entering the farm and again before getting into the vehicle (45). Moreover, parking areas should be designed to avoid cross-contamination between workers and farm vehicles. In case vehicles have to enter into the farm, loading and unloading areas should be placed at least 20 m away from animal facilities within the perimeter of the farm (66). Vehicles transporting pigs and other vehicles must be cleaned and disinfected before and after each use (42, 45). Returning trucks should be cleaned and disinfected at the farm where pigs are unloaded (45). In addition to this, the Danish regulation applies a 48 h quarantine period before the next movement of animals (27). After that new animals should be kept in quarantine rooms (16, 35, 36, 55) between 14 and 30 days (5, 18, 45, 58, 64). Quarantine rooms should be located away from the main herd (45).

Furthermore, animals should be identified and all animal movements recorded (15, 23, 32, 45, 86); farm records should be ensured to easy track animals if an outbreak is reported; births and deaths, animal census, entry and exit of animals (live and dead), vehicles, visits, pest control, or cleaning and disinfection procedures should be properly registered in a farm record book (16, 55, 65). Moreover, internal audits or self-evaluation need to be periodically conducted to enforce biosecurity measures (15, 18). Furthermore, a set of rules on food for workers entering the farm should be clearly specify (31) and food should be restricted to eating rooms (15, 18) or not allowed (44, 52).

Finally, proper disposal of manure as well as dead animals and other removable material should be ensured (5, 58, 65). Containers and storage basins should accomplish with the minimum requirements for storage capacities recommended by the Best Available Techniques (71).

#### Specific Measures Focusing on Non-Commercial Farms

Backyard farms are characterized by limited farming management practices and nearly absent biosecurity levels (5, 6, 55). This type of farm is common in countries such as Romania (46), Bulgaria (80), Poland (87), or Sardinia (Italy) (6, 23), among others. Non-commercial farms are built for own consumption purposes, investment is minimum and animals could be fed on kitchen leftovers (88). Depending on the country and local practices, pigs are allowed to move freely (without physical restrictions) during the day or even scavenge for days or months (46, 74, 89). Pig slaughtering is usually carried out on the farm, although it may be restricted to proper slaughterhouses if there are local regulations on this issue (16, 46).

Specific measures focusing on these farms have been proposed, swill feeding practices are not allowed (15, 22, 43, 45, 47, 52, 57), as ASF can be transmitted through ingestion of contaminated raw pork or pork products (5, 15, 38, 64). Pigs should be kept in animal facilities ensuring no contact with domestic pigs from other non-commercial farms, feral pigs, wild boar nor their products (5, 15, 43, 55). If there were infected wild boar in the area, the owner or the person in charge of taking care of the pigs should allow a 48 h interval between hunting and being in contact with domestic pigs (15, 61) and should not use dogs during hunting (61). Any hunting equipment used as well as the dog’s coat should be cleaned and disinfected (42). Effective disinfectants such as calcium hydrate (slaked lime), should be spread and renewed around the holding including its entrance (5). A veterinarian needs to supervise home slaughtering activities (15, 72). If a slaughterer comes to slaughter the animals, cleaned and disinfected clothing and footwear should be provided. Cleaning and disinfection protocols have to be applied after slaughtering on the facilities and to the slaughter tools (15, 16). The Directorate-General for Health and Food Safety and the Sardinian regulations agree that sows or boars cannot be held on non-commercial farms for mating purposes (15, 72) while Decision 830/2016 of the Romanian Government states that sows and boars might be present but they cannot be moved between holdings for matting purposes (43), movements from these farms are neither allowed in the Sardinian regulations (72). Furthermore, governments and institutions are encouraged to promote educational programs as well as improve access to health services on non-commercial farms (4, 15, 23). This measure is one of the novelties of the latest eradication program launched in Sardinia (20).

Moreover, the use of fresh fodder harvested in areas at risk for ASFV exposure should be avoided (15, 28, 53), as its consumption has been observed that could be related to ASF outbreaks in Eastern EU countries (53). If this is not possible, Directorate General for Health and Food Safety (15) recommends to perform treatments on grass or grains to inactivate ASFV or store them, out of reach of wild boar, for at least 30 days. In Estonia, according to the Regulation of the Minister of Agriculture No. 179, it is forbidden to bring green fodder to the farm (47). Likewise, Directorate General for Health and Food Safety (15) recommends to avoid using straw as bedding material unless treated to inactivate ASFV or stored for at least 90 days (15). Additionally, the Estonian Veterinary and Food Board established as compulsory biosecurity rule, no exchange feed and bedding material with other farms (47). Field experiences showed that no additional cases were reported when non-commercial farm had feed from reliable sources and contact with infectious free-ranging pigs was prevented (55).

#### Specific Measures Focusing on Outdoor Farms

The number of outdoor farms is increasing in Europe due to a growing interest in organic farming systems (90), particularly from pork consumers due to animal welfare concerns. Simultaneously, veterinarians and pig producers have been urging for improvements in biosecurity, so as to avoid health threats (91). Depending on the country and local practices, outdoor pig production may vary from outdoor farms that implement several biosecurity measures (92), to free-ranging herds where biosecurity is absent (6).

Spain is a good example of a country with strict biosecurity standards for outdoor pig production. Regulations regarding biosecurity on outdoor pig farms (73) are a result of the presence of ASF for more than 30 years in the Iberian Peninsula (65). Applied control and preventive measures allowed to eradicate ASF from outdoor pig production and avoided new introductions on outdoor farms, despite the constant threat posed by the presence of infected wild boar and infectious *Ornithodoros* ticks in the surroundings (4, 65). In other areas such as Sardinia in Italy, pigs are allowed to range free in public forests during the day, for days or even months under no biosecurity measures (41). Free-range management practices in communal areas has been identified as a dangerous practice for the persistence and re-emergence of ASF in endemic areas like Sardinia (6, 16, 74). During the free-ranging period, pigs might be in contact with wild boar and pigs belonging to different herds (30, 32, 89). For this reason, free-range management practices in communal areas or public forest with no biosecurity measures nor veterinary control have been banned (5, 16, 20, 23), such as in Sardinia since 2012 (20).

Bearing in mind the current situation in Eastern Europe, the EU Commission has banned outdoor keeping of pigs as the main strategy to avoid ASF spread (15, 47). Although prevention becomes challenging in outdoor and semi-extensive pig production (74), several preventive measures can be implemented to ensure biosecurity levels. For instance, the territories/fields where animals are allowed to range free should be fenced (double fenced, if it is possible) to avoid the entrance and direct contact with wild boar, feral pigs, and other domestic pigs, as well as people and vehicles (5, 42, 49, 55). Sardinian regulations state farms should have perimeter barriers of at least 1.5 m high and wild boar proofed and fenced fields had a maximum extension of 3 ha (72). Outdoor farms should be separated from other outdoor farms to reduce the risk of ASF introduction through direct or indirect contact (73). This minimum distance between farms will vary depending on national and local regulations. If pigs were free to roam within no fenced fields, distance would become irrelevant (18).

So far, *Ornithodoros* ticks have not been implicated in the transmission of ASF in Eastern nor Central Europe (64). In Sardinia, ticks have also not been identified as a major transmission source (93). Several preventive measures were described in Portugal and Spain were *Ornithodoros erraticus* are present such as keeping traditional pig-housing facilities (typically, used in outdoor production), in good repair, otherwise it is recommended to fence them or destroy them if ticks are present (34, 64, 65). In case ticks are present, either chemical control with methylene bromide should be applied on the facilities, or treating pigs with an ivermectin treatment (34). If infected ticks were present in such constructions, it is not recommended to use the infested buildings (54) or keep these buildings empty for 6 years (19). Nevertheless, it should be considered that eradication of *O. erraticus* ticks is extremely difficult due to the long life of ticks, long survival without feeding, presence of accidental hosts, and possibility of penetrating into cracks and surfaces not accessible to acaricides (54).

Table 2 compiles the general preventive measures and specific preventive measures for commercial, non-commercial, and outdoor farms described in this review.

**Table 2:** General measures to prevent African swine fever spread on domestic pig farms plus specific measures focused on commercial (CM), non-commercial (NCM), and outdoor holdings (OD).

ID	Preventive measures	Systematic literature review		Results of the assessment		
		Type	Reference	CM	NCM	OD
1	Check ASF-free certificates and health status before acquiring new animals as well as semen, ova or embryos on breeding farms	General (CM, NCM, OD)	(15, 16, 18, 19, 21, 40, 44, 45, 57, 58, 63, 64)	Yes: 100%	Yes: 83% No: 17%	Yes: 100%

2	Limited farm visitation with proper register and establishment of biosecurity measures regarding footwear and clothing	General (CM, NCM, OD)	(5, 31, 44, 45, 47, 52, 57, 58, 63, 64)	Yes: 100%	Yes: 83% No: 17%	Yes: 92% No: 8%

3	Farmers/workers and operators education	General (CM, NCM, OD)	(22, 23, 37, 38, 47, 48, 52, 57, 58, 63)	Yes: 100%	Yes: 92% No: 8%	Yes: 100%

4	Farmers/workers should not contact with external pigs	General (CM, NCM, OD)	(4, 5, 15, 16, 57)	Yes: 92% No: 8%	Yes: 92% No: 8%	Yes: 92% No: 8%

5	Perimeter fences to prevent contacts with external pigs and wild boar	General (CM, NCM, OD)	(15, 16, 18, 45, 47, 55, 57, 63, 65, 66)	Yes: 92% No: 8%	Yes: 67% No: 33%	Yes: 100%

6	Appropriate removal of carcasses, slaughter residues and food waste	General (CM, NCM, OD)	(57, 58, 63, 67)	Yes: 100%	Yes: 92% No: 8%	Yes: 100%

7	Discouragement of sharing used equipment between holdings and/or units	General (CM, NCM, OD)	(45, 47, 52, 55, 57, 63)	Yes: 100%	Yes: 83% No: 17%	Yes: 100%

8	Use of footbaths in entrance of units where animals are held	General (CM, NCM, OD)	(5, 15, 49, 52, 58, 63)	Yes: 75% No: 25%	Yes: 50% No: 50%	Yes: 33% No: 67%

9	Daily health checks for clinical signs and mortality rates	General (CM, NCM, OD)	(45)	Yes: 100%	Yes: 58% No: 42%	Yes: 92% No: 8%

10	Cleaning and disinfectant protocols for facilities, vehicles, and equipment	General (CM, NCM, OD)	(15, 16, 18, 35, 42, 44, 49, 55, 57, 63)	Yes: 100%	Yes: 50% No: 50%	Yes: 92% No: 8%

11	Farm location far from suitable wild boar areas and close to geographical barriers	General (CM, NCM, OD)	(16, 28, 48, 53)	Yes: 67% No: 33%	Yes: 58% No: 42%	Yes: 75% No: 25%

12	Control measures against flies	General (CM, NCM, OD)	(45, 68, 69)	Yes: 75% No: 25%	Yes: 42% No: 58%	Yes: 17% No: 83%

13	Establishing clean/dirty areas (including changing rooms and showers)	CM	(15, 18, 31, 49, 55)	Yes: 92% No: 8%	Yes: 58% No: 42%	Yes: 67% No: 33%

14	Logistical arrangement for the entry and exit of animals including protocols regarding entrance of vehicles, loading areas, role of pig transporters, etc.	CM	(5, 15, 18, 42, 45, 58, 65, 66, 70)	Yes: 100%	Yes: 33% No: 67%	Yes: 75% No: 25%

15	Cleaning and disinfection protocols for transport vehicles	CM	(27, 42, 45)	Yes: 100%	Yes: 58% No: 42%	Yes: 83% No: 17%

16	Quarantine period for purchased animals and quarantine rooms	CM	(5, 16, 18, 24, 35, 45, 55, 58, 64)	Yes: 100%	Yes: 25% No: 75%	Yes: 67% No: 33%

17	Identification of animals and farm records including animal movements	CM	(15, 16, 23, 27, 32, 45, 55, 65)	Yes: 100%	Yes: 100%	Yes: 100%

18	Internal audits and evaluations to enforce biosecurity measures	CM	(15, 18)	Yes: 92% No: 8%	Yes: 25% No: 75%	Yes: 67% No: 33%

19	Rules for food staff entering the farm (i.e., restricted to eating rooms or not allowed)	CM	(15, 18, 44, 52)	Yes: 100%	Yes: 58% No: 42%	Yes: 75% No: 25%

20	Proper disposal of manure and dead animals	CM	(5, 58, 65, 71)	Yes: 100%	Yes: 92% No: 8%	Yes: 92% No: 8%

21	Strict enforcement of the ban on swill feeding	NCM	(5, 15, 22, 38, 43, 45, 47, 52, 57, 64)	Yes: 100%	Yes: 100%	Yes: 100%

22	Containment of pigs, do not allow contact with pigs from other farms, feral pigs, or wild boar or their products	NCM	(5, 15, 43, 55)	Yes: 100%	Yes: 100%	Yes: 100%

23	Farmers/farm staff should not have hunted, allowing a 48 h interval between hunting and being in contact with domestic pigs, if they work in an infected wild boar area	NCM	(15, 61)	Yes: 92% No: 8%	Yes: 92% No: 8%	Yes: 92% No: 8%

24	Effective disinfection and cleaning of the surrounding of the holding including its entrance	NCM	(5)	Yes: 58% No: 42%	Yes: 50% No: 50%	Yes: 42% No: 58%

25	Veterinary supervision prior and while home slaughtering	NCM	(15, 72)	Yes: 50% Na: 33% No: 17%	Yes: 83% No: 17%	Yes: 67% Na: 16.5% No: 16.5%

26	Cleaning and disinfection protocols before and after home slaughter (regarding slaughtering tools, facilities, clothing and footwear, etc.)	NCM	(15, 16)	Yes: 42% Na: 25% No: 33%	Yes: 83% No: 17%	Yes: 67% Na: 8% No: 25%

27	No sows or boars used for mating purposes held on non-commercial farm	NCM	(15, 72)	Yes: 58% Na: 42%	Yes: 67% Na: 8% No: 25%	Yes: 42% Na: 33% No: 25%

28	No movements between/from non-commercial farms	NCM	(43, 72)	Yes: 75% Na: 17% No: 8%	Yes: 92% No: 8%	Yes: 67% Na: 16.5% No: 16.5%

29	Avoid use of fresh fodder in areas at risk of exposure to ASF	NCM	(15, 22, 28, 47)	Yes: 67% No: 33%	Yes: 75% No: 25%	Yes: 75% No: 25%

30	Promote educational programs through governmental training programmes and improve access to health services	NCM	(4, 15, 20, 23)	Yes: 92% No: 8%	Yes: 100%	Yes: 100%

31	Treatment and storage (out of reach of wild boars) of grass or grains for at least 30 days or prohibit its use	NCM	(15, 47)	Yes: 75% No: 25%	Yes: 75% No: 25%	Yes: 67% No: 33%

32	Avoid the use of straw bedding unless treated to inactivate ASF and stored for at least 90 days	NCM	(15)	Yes: 83% No: 17%	Yes: 83% No: 17%	Yes: 83% No: 17%

33	No exchange of feed or bedding with other farms	NCM	(47)	Yes: 92% No: 8%	Yes: 92% No: 8%	Yes: 92% No: 8%

34	Banning of free-range management on communal areas or public forests with no biosecurity measure	OD	(5, 15, 16, 20, 23, 32, 47)	Yes: 67% Na: 16.5% No: 16.5%	Yes: 92% No: 8%	Yes: 92% No: 8%

35	Distance between outdoor farms (at least 1 km) to minimize the risk of ASF introduction through direct and indirect contact	OD	(73)	Yes: 16% Na: 42% No: 42%	Yes: 33% Na: 25% No: 42%	Yes: 67% No: 33%

36	If they were *Ornithodoros* ticks avoid using traditional pig-housing facilities (usually made of wood and stones were ticks can be hidden)	OD	(34, 54, 64, 65)	Yes: 84% Na: 8% No: 8%	Yes: 75% Na: 8% No: 17%	Yes: 84% Na: 8% No: 8%

37	Apply chemical control if ticks were present in traditional pig-housing facilities	OD	(34)	Yes: 84% Na: 8% No: 8%	Yes: 92% Na: 8%	Yes: 92% Na: 8%

*Results of the assessment of identified preventive measures represented as percentage of yes, not applicable (Na) and no*.

### Assessment of the Importance of Described Preventive Measures

A total of 12 experts participated in the assessment of the importance of identified preventive measures. All of them completed the questionnaire and therefore, their responses were included in the analysis. Around 3% of assessed measures (2.85%) were categorized as “not applicable” preventive measure.

There was 100% agreement among experts (12 experts out of 12) that the identification of animals and farm records including animal movements; enforcement of the ban on swill feeding; and containment of pigs to not allow contact with pigs from other farms, feral pigs, or wild boar or their products, were important preventive measures for the three types of farms (commercial, non-commercial, and outdoor). Other important preventive measures identified for all farms were education of farmers, workers, and operators; no contact between farmers and farm staff and external pigs; appropriate removal of carcasses, slaughter residues and food waste; proper disposal of manure and dead animals; and a 48 h (minimum) interval between hunting and being in contact with domestic pigs for all farm staff, particularly those who work in an infected wild boar area.

Moreover, all experts identified as important preventive measures for non-commercial and outdoor farms, to improve access of those farms to veterinarians and health services. Between eight and nine of experts considered that logistical arrangement for the entry and exit of animals including protocols regarding entrance of vehicles, loading areas and role of pig transporters; quarantine period for purchased animals and quarantine rooms; and internal audits and evaluations to enforce biosecurity measures, were not important preventive measures for non-commercial farms. In addition, 10 experts concluded that control measures against flies were not an important preventive measure on outdoor farms.

Additional preventive measures were suggested by some experts such as the use of nets on animal facilities; establishment of pest control programs on farms; use of carbonic dioxide traps to check the presence of *Ornithodoros* ticks and change of boots before entering the farm and units. Furthermore, several respondents wanted to emphasize the importance of measures already included in the questionnaire. For instance, establishment of double fencing perimeter on outdoor farms; education of swine veterinarians and farmers paying especial attention to clinical signs and transmission routes; and discouragement of using the same injection syringes and instruments on different farms unless thoroughly disinfected sterilized.

**Figure 2:**
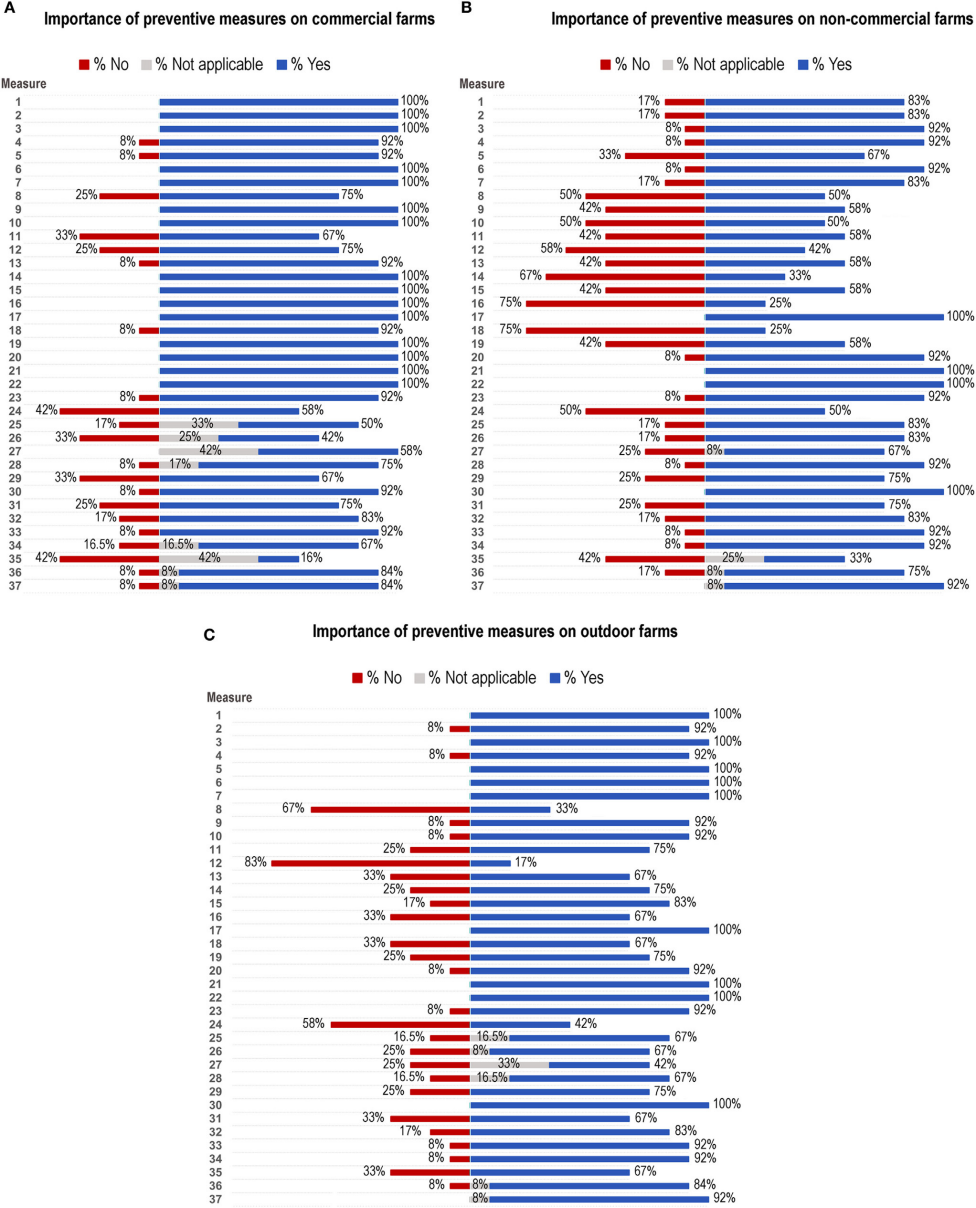
Results of the assessment of identified preventive measures represented as percentage of yes (blue bars), no (red bars), and not applicable (gray bars) to **(A)** commercial farms, **(B)** non-commercial farms, and **(C)** outdoor farms. Listed preventive measures are described in Table 2.

Figure 2 and Table 2 summarize the results obtained for preventive measures on commercial, non-commercial, and outdoor farms.

## Discussion

In the absence of an effective vaccine, prevention is the main tool to avoid further spread of ASF or an endemic situation. Both the systematic literature review as well as the expert opinion elicitation, highlighted three main areas where preventive measures would be very relevant to halt ASF spread in the domestic pig population: (1) control of entries into the farm, (2) control of pigs’ feed, and (3) improvement of health services and education.

The first main area of prevention encompasses both the movements associated to production as well as the potential spill-over from infected wild boar in the surrounding areas. Both have been major drivers of spread in the current ASF epidemic in Eastern EU, where the majority of ASF notifications in domestic pigs have occurred in backyard or small commercial farms with limited biosecurity (11). The identification of animals and the containment of pigs were also identified by the experts as important preventive measures for all type of holdings. Quarantine period for purchased animals in quarantine rooms was identified as a relevant measure for commercial farms by 12 experts. In agreement with this result, experts in Switzerland perceived that purchasing from farms with known disease status and health certificates as 5/5 for importance and effectiveness as a biosecurity measure to prevent the introduction of ASF onto pig farms (29). Interestingly, 9 experts out of 12 did not consider this measure important on non-commercial farms, although the same number consider it important to check ASF-free certificates and health status before acquiring new animals. This may be explained because the feasibility of quarantine periods and establishment of quarantine rooms and procedure could be challenging on non-commercial farms where investment and facilities are minimum. This measure becomes particularly relevant when tackling the phenomenon of “emergency sale,” in which farmers from non-commercial holdings attempt to sell infected pigs to minimize their economic losses (87, 94–96). The latest working document elaborated by the Directorate General for Health and Food Safety (15), which contains the majority of measures reviewed in the systematic literature review, aim at the improvement of biosecurity measures dealing with the replacement of animals, facilities design, and management practices, in particular in relation with cleaning and disinfection facilities, in such holdings. Very few outbreaks have led to secondary spread in the EU and there has been a significant progress in EU advice to improve preventive measures against ASF in non-commercial farms.

The Eastern EU scenario presents the additional challenge of spill-over from wild boar, where 95% of the ASF notifications have taken place (8) and which is playing a primary role in disease spread. However, additional measures were extracted during the review process (see Table 1). In Poland and Latvia, outbreak investigations carried out on several ASF positive farms determined that the most likely source of infection was wild boar (82, 97). Studies concluded that the poor biosecurity measures of affected holdings favored transmission between wild boar and domestic pigs (82, 97). Consequently, the EU elaborated a guidance where minimum biosecurity measures on farms were defined and biosecurity was enhance to minimize the risk of spread from wild boar (15, 63). One of the suggested measures found in the literature is “to locate farms far from suitable wild boar areas and close to physical barriers” (16, 48, 53, 83) since there is a disease interface where domestic pig and wild boar share location. Observations related to the wild boar–domestic pig interface indicated that all ASF notifications in domestic pig holdings were situated in areas with suitable wild boar habitat (53). Around 65% occurred in natural landscapes, the natural habitat for wild boar (28). The remaining 35% were located in mosaic agroforestry areas and buffer monoculture areas surrounding natural landscapes where agro-livestock activities are usually concentrated (28). In these areas, wild boars can receive, with minimal foraging, substantial amounts of protein from cultivated plants such as maize, wheat, barley, rapeseed, and sunflower seeds (98). Farm location far from suitable wild boar areas and close to geographical barriers was classified as important by more than half of experts. As expected, such measures were relevant to more experts on outdoor farms (9 experts), followed by commercial (8 experts), and non-commercial holdings (7 experts). This slight difference might be explained because the likelihood of wild boar being in contact with pigs would be higher on outdoor farms (where biosecurity is intrinsically lower) than on commercial or non-commercial farms. Experts who declined to consider it important, refereed that this measure is almost unfeasible considering the ecological characteristics of the European continent. Moreover, some of the experts who considered it important wanted to highlight that such a measure would only be applicable to new holdings.

Most experts (11 out of 12) recognized the importance of allowing a 48 h interval between hunting and being in contact with domestic pigs if farmers and farm staff worked in an infected wild boar area. Although it is not the scope of this article to cover the control measures in wild boar, the management of wild boar populations and hunting practices in affected areas has an undeniable effect over the prevention of ASF at the interface with domestic pigs located in the same area. Such measures have included the reduction of wild boar densities (53, 59) and the immediate removal of infectious carcases (5). However, wild boar cases have continued being notified in the area suggesting that there is still room for improving the strategy.

The second main area of prevention deals with avoiding ASF transmission through the ingestion of contaminated food. Even if swill feeding is banned in the EU, all experts agreed that it was an important measure to prevent ASF spread. Other measures identified in this sense are rules on food entry for farm workers in commercial farms; proper disposal of manure and dead animals; avoiding the use of fresh fodder from areas at risk of ASF unless a treatment to inactivate potential ASF virus, has been applied; or avoid sharing feed between farms. Long distance ASF transmission has been associated to the disposal of infected waste, meat or meat products in wild boar habitat, for example, in the Czech Republic, where the closest ASF cases were about 400–500 km away. Moreover, evidences of domestic pigs and/or pig sub-products as source of infection are scarce but they have been suspected in a few cases, like in Romania. On July 31, 2017, Romania’s Veterinary Authority confirmed the first detection of ASF in a backyard herd of domestic pigs. Romania’s Veterinary Authority suspects that contaminated Ukrainian products are the likely source of the Romanian detection (99). Human mistakes, lack of knowledge on ASF transmission, or insufficient enforcement are the most common reasons to fail to comply with these measures, particularly for non-commercial farms, and are directly related to the third main area of ASF prevention: improvement of health services and education.

Better access to veterinary health services and educational programmes, with specific training on ASF identification and biosecurity measures, are essential tools to improve human-mediated prevention measures. More than 11 experts agreed with this idea, considering both measures important for non-commercial farms but also, for commercial and outdoor facilities. In the end, effectiveness of prevention depends on awareness, compliance and diligence of people dealing with disease control and good timing of implemented measures (100). The effectiveness of prevention is also influenced by socioeconomic, cultural, or traditional factors that will predispose the capability, attitudes, or willingness of people involved in disease control to implement preventive strategies. The understanding of such factors is particularly critical for backyards and small farmers, since economic and resources restraints can more easily limit the achievement of the preventive measure objective (16, 95). Generally, the effectiveness of preventive measures will be related to how farmers perceive the importance of each measures as well as what measures they are actually implementing (55). Farmers and workers are at the forefront of implementing biosecurity measures on the farms to prevent the spread of diseases. The application of these measures heavily depends upon the attitude and knowledge they have with regard to biosecurity measures (101). A study carried out in Great Britain showed that English pig farmers had poor knowledge about ASF as well as limited concern about it (26). Vergne et al. (102) also highlighted that the reasons for lack of immediate reporting in suspected ASF cases in Germany, the Russian Federation, and Bulgaria would be due to not knowing reporting procedures, fear that the report could have a negative impact on their reputation, and assuming they would be capable of handling the outbreak on their own. These studies (26, 102, 103) suggested that there is still room for improving farmers’ knowledge to bridge the gap between authorities and farmers and consequently help prevent the spread of ASF (39). Similarly, to be able to effectively influence farm workers, veterinarians, and hunters’ behavior, it is essential to analyze the “at-risk” practices that depended on human behavior which can perpetuate ASF spread and find out measures tailored to each specific situation.

From the research side, efforts have been made to fill in gaps that make disease control and eradication difficult. A recent publication identified current gaps in ASF and prioritized them into high importance, medium importance, and low importance (48). Highest importance was attributed to measures aimed at improving prevention and control of ASF, namely, (i) to raise awareness among hunters, farmers and veterinarians and (ii) to have adequate implementation of early warning systems, contingency plans, and control measures. Preventive measures of medium importance were (iii) to implement surveillance activities based on the risk of potential exposure, introduction and spread. Measures of low importance were (iv) to promote confinement of pigs in infected areas, and (v) to establish regulations to ensure farms are located far from areas suitable for wild boar. Finally, with regard to the importance of wild boar in ASF epidemiology, more research should be focused on (vi) increasing the availability of reliable population data, (vii) understanding role of this host in disease maintenance and spread, and (viii) developing non-invasive sampling methods (48, 50, 59). However, without an ASF vaccine, prevention of ASF becomes very challenging for the European pig sector. Despite advances, a safe and effective vaccine is still lacking. Thus, control and eradication of this disease still relies on rapid detection in field followed by the application of strict sanitary measures. Likewise, biosecurity is the only tool farms have to prevent the introduction of ASF. Therefore, joined efforts focusing on the domestic pig sector and wild boar need to be applied in parallel. This way, we will move forward to the final goal of eradicating ASF from the second largest world’s pork producer, the EU.

## Conclusion

African swine fever is currently one of the major threats to the pig production in the EU. As there is no a vaccine against ASF, biosecurity is key to prevent its spread between and within domestic pig farms. This study identified thirty-seven preventive measures aimed at reducing the spread of ASF among domestic pigs. These measures were also assessed by ASF experts within the framework of the EU scenario. According to this expert panel, the most important preventive measures for commercial, non-commercial, and outdoor farms were the identification of animals and farm records; enforcement of the ban on swill feeding; and containment of pigs to not allow contact with pigs from other farms, feral pigs, or wild boar or their products. In addition to this, other measures were considered relevant in preventing ASF introduction, namely education of farmers, workers, and operators; no contact between farmers, farm staff and external pigs; appropriate removal of carcasses, slaughter residues and food waste; proper disposal of manure and dead animals, and abstention from hunting activities for a period of 48 h prior to any contact with domestic pigs. Finally, all experts considered important to facilitate and promote the access of veterinarians and health services to non-commercial and outdoor farms. Adequate implementation of these measures can lead to significant advances in ASF prevention and control, and possibility contributing to the eradication of ASF from the EU pig sector.

## Author Contributions

All authors contributed to the literature review performed to build this review. CJ compiled the whole information and wrote the manuscript. CJ, JS-V, and SB designed the questionnaire for the assessment of preventive measures. CJ analyzed results from the expert opinion. CJ, SB, JS-V, MM-A, and AT participated in the creation of the argument line of this the text. All authors contributed to the critical review of the manuscript and approved the final version.

## Conflict of Interest Statement

HF was employed by company GD Animal Health Research Institute. All other authors declare no competing interests. The reviewer AB and handling Editor declared their shared affiliation.

## Footnotes

^1^http://www.ncbi.nlm.nih.gov/pubmed (Accessed: March 3, 2017).

^2^https://www.asf-stop.com/stsms/ (Accessed: December 14, 2017).
